# Application of augmented reality technology for obstetrics and gynecology teaching among undergraduates

**DOI:** 10.1186/s12909-025-07751-1

**Published:** 2025-08-08

**Authors:** Xuan Rao, Qian-Qian Wu, Jin Yang, Xiao Li

**Affiliations:** 1 Department of Gynecologic Oncology, Women’s Hospital, School of Medicine, Zhejiang University, No.1 Xueshi Rd, Hangzhou, Zhejiang 310006 China; 2 Zhejiang Provincial Key Laboratory of Precision Diagnosis and Therapy for Major Gynecological Diseases, Hangzhou, Zhejiang China

**Keywords:** Augmented reality, Obstetrics and gynecology, Anatomical teaching, Medical undergraduates.

## Abstract

**Objective:**

The anatomy of the female reproductive system is the foundation of obstetrics and gynecology (OBGY) education. Augmented reality (AR) is now emerging information technology, which has been increasingly used in medical education of other disciplines. The present study aimed to evaluate whether AR-assisted learning mode could enhance the spatial comprehension and clinical thinking ability of undergraduates during the OBGY teaching.

**Methods:**

Medical undergraduates undergoing clinical studies were enrolled. Initially, all students received traditional classroom teaching with the textbook. Subsequently, they were directed to study identical curricular content through AR software in a self-guided online format. The value of AR technology for OBGY teaching was assessed via a self-report questionnaire following completion of AR-assisted learning.

**Results:**

In total, 285 valid questionnaires were collected. The AR-assisted learning mode was accepted by 99.30% students. Most students (98.60%) believed that AR-assisted learning can not only enhance their understanding of the 3D anatomy, but also help to cultivate their clinical thinking ability. Interestingly, compared with pre-class AR-assisted learning, more students accepted post-class learning (26.32% vs.73.68%). To our surprise, almost all students (98.95%) gave a positive evaluation of the AR-assisted learning mode in our study. Compared with the traditional teaching mode, we found that top advantages of AR-assisted learning mode were clearer, more intuitive and vivid (56.14%). Other perceived benefits included enhanced understanding of the spatial structure (9.47%), convenience and not limited by time and place (2.81%), and increased learning interest (2.11%).

**Conclusions:**

This study is the first to demonstrate that AR technology is a valuable supplementary tool to be integrated into traditional OBGY teaching for medical undergraduates.

**Supplementary Information:**

The online version contains supplementary material available at 10.1186/s12909-025-07751-1.

## Introduction

Virtual reality (VR) and augmented reality (AR) have recently emerged as innovative information technologies. VR technology generates a three-dimensional (3D) visual image through the computer, allowing users to engage deeply with virtual scenarios to obtain comprehensive information [1, 2]. Although AR is similar to VR in many aspects of the technology, there are some essential differences. For example, the purpose of AR is not to build a completely virtual environment, but to overlay the computer-generated images on the real-world images and enhance the user’s real experience [3–5]. In recent years, AR technology has been increasingly applied in medical education such as anatomy teaching, clinical skill training and surgical training, especially in cardiology, neurosurgery and stomatology [6–11]. Cumulative evidence demonstrates that AR technology significantly improves learning efficiency, enhances spatial understanding ability, and strengthens clinical operation skills [12–17]. However, its integration into the obstetrics and gynecology (OBGY) teaching remains underexplored.

The anatomy of the female reproductive system forms the cornerstone of OBGY education, yet its complexity and privacy often hinder medical undergraduates’ comprehension. This is particularly evident in teaching the delivery mechanism, where students must interpret 3D spatial dynamics between the fetus and maternal pelvis from 2D pictures-a process requiring advanced spatial visualization skills. Moreover, students have limited opportunities to observe various clinical operations of OBGY during the undergraduate teaching procedure. Due to the high demand for imaginative ability in the traditional teaching mode, it is undoubtedly difficult to stimulate learning enthusiasm and achieve the ideal learning effect. Therefore, developing innovative approaches to vividly convey key OBGY knowledge and enhance clinical practice competency would be a pressing challenge in modern medical education.

The present study aimed to evaluate whether an AR-assisted learning mode could promote the independent learning and clinical thinking abilities of undergraduates during OBGY teaching by self-report questionnaires of students’ subjective evaluations following completion of AR-assisted learning, compared with traditional classroom teaching mode. Furthermore, the students’ feedback on AR-assisted learning mode was collected to inform future refinements of both the technology and teaching methodologies.

## Materials and methods

### Participants

A total of 312 clinical medicine undergraduates undergoing clinical studies in the Women’s Hospital School of Medicine Zhejiang University between April 25 and May 25, 2022 were included in the present study. It was approved by the Ethics Committee of the hospital (IRB-20220314-R). Given that study data were collected via anonymous questionnaires, informed consent was specifically waived by the ethics committee.

### Teaching methods

The ninth edition of OBGY textbook, nationally designated for five-year medical undergraduates, served as the teaching material. The normal anatomy of the female reproductive system and delivery mechanism sections were selected as the teaching content in the present study. The AR learning software was affiliated with OBGY textbook, which could be downloaded by users with mobile devices. By scanning the 2D anatomical images in the book using this software, 3D, dynamic and interactive anatomical images of the female reproductive system and delivery process will appear directly on the mobile devices. It supports operations such as zooming in or out, rotating, and labeling, allowing learners to comprehensively understand anatomical details from multiple angles.

Initially, all students received traditional classroom teaching with the textbook. Subsequently, they were directed to study the 3D anatomy of the female reproductive system and the delivery process through AR software in a self-guided online format.

### Teaching evaluation

The value of AR technology for OBGY teaching was assessed via a self-report questionnaire following completion of AR-assisted learning (Table 1). The questionnaire evaluated dimensions including usage duration, learning interest and AR-assisted learning value.

**Table 1:** Questionnaire provided to the students, containing explicit statements and evaluation about AR-assisted learning mode

Questions	Answers					
How long did it take you to learn the course on anatomy of the female reproductive system by AR? (minute)	5–10	11–15	16–20	21–25	26–30	>30
How long did it take you to learn the course on anatomy of delivery process by AR? (minute)	5–10	11–15	16–20	21–25	26–30	>30
You prefer AR-assisted learning mode	Strongly agree	Agree	Acceptable		Disagree	Strongly disagree
AR helps stimulate learning interest	Strongly agree	Agree	Acceptable		Disagree	Strongly disagree
AR helps promote active learning	Strongly agree	Agree	Acceptable		Disagree	Strongly disagree
AR helps understand the three-dimensional spatial structure	Strongly agree	Agree	Acceptable		Disagree	Strongly disagree
AR helps improve memory of learning content	Strongly agree	Agree	Acceptable		Disagree	Strongly disagree
AR helps train clinical thinking	Strongly agree	Agree	Acceptable		Disagree	Strongly disagree
Do you think there is a difference in the learning effect between the AR-assisted pre-class and post-class learning?	Pre-class learning is better		No difference		post-class learning is better	
Your overall evaluation of AR-assisted learning mode	Excellent	Good	Acceptable		Poor	Unacceptable
Rating your satisfaction with the course on anatomy of the female reproductive system (points)	<60 (Not satisfied)	60–69 (Average)	70–79 (Good)		80–89 (Satisfied)	90–100 (Very satisfied)
Rating your satisfaction with the AR-assisted course on anatomy of the female reproductive system (points)	<60 (Not satisfied)	60–69 (Average)	70–79 (Good)		80–89 (Satisfied)	90–100 (Very satisfied)
Rating your satisfaction with the course on anatomy of delivery (points)	<60 (Not satisfied)	60–69 (Average)	70–79 (Good)		80–89 (Satisfied)	90–100 (Very satisfied)
Rating your satisfaction with the AR-assisted course on delivery (points)	<60 (Not satisfied)	60–69 (Average)	70–79 (Good)		80–89 (Satisfied)	90–100 (Very satisfied)
Write down the advantages and disadvantages of AR-assisted learning mode						
Write down the suggestions for improvement of AR-assisted learning mode						

### Statistical analysis

All statistical analyses were performed using the SPSS 25.0 Statistics (IBM Corp.). Questionnaire responses were summarized as percentage (%). Pearson’s or continuous corrected χ2 test was used to compare qualitative data. All *p*-values were two-sided and *p* < 0.05 was considered statistically significant.

## Results

### Learning interest stimulated by AR technology

In total, 285 valid questionnaires were collected from 312 students (response rate: 91.35%). For the anatomy of female reproductive system, most students (78.95%) used AR technology within 20 min, while only 5.61% of students used AR technology for more than 30 min. A similar pattern was observed in the normal delivery process (83.16% vs. 3.61%).

**Fig. 1:**
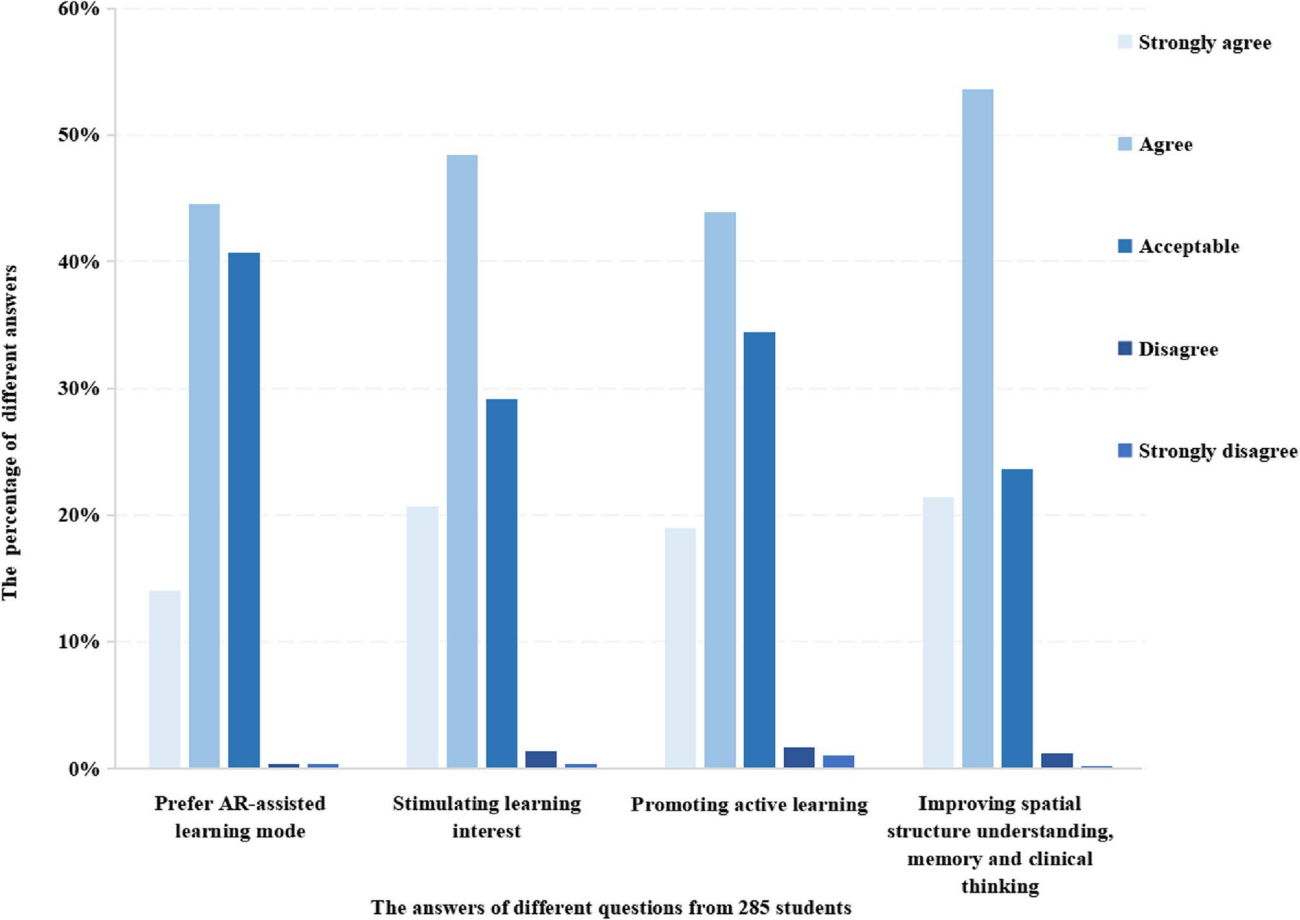
The distribution of different answers of six questions from 285 students. Six questions included if “You prefer AR-assisted learning model”, “AR helps stimulate learning interest”, “AR helps promote active learning”, “AR helps to understand the three-dimensional spatial structure”, “AR helps improve memory of learning content” and “AR helps train clinical thinking”. Specifically, the answers to the last three questions were merged together into one class for analysis and comparison

As shown in Fig. 1, the AR-assisted learning mode was accepted by 99.30% of students. Over 95% of students agreed that AR technology could stimulate learning interest (strongly agree: 20.70%, agree: 48.42%, acceptable: 29.12%) and promote active learning engagement (strongly agree: 18.95%, agree: 43.86%, acceptable: 34.39%).

### Value evaluation of AR-assisted learning mode

Compared with the traditional teaching mode, A key advantage of AR-assisted learning mode is its ability to visualize 3D anatomical structures, enabling multi-perspective comprehension. As shown in Fig. 1, students strongly endorsed this benefit. Specifically, most students (98.60%) agreed that AR-assisted learning improved their understanding and retention of 3D anatomy while fostering clinical thinking ability.

**Fig. 2:**
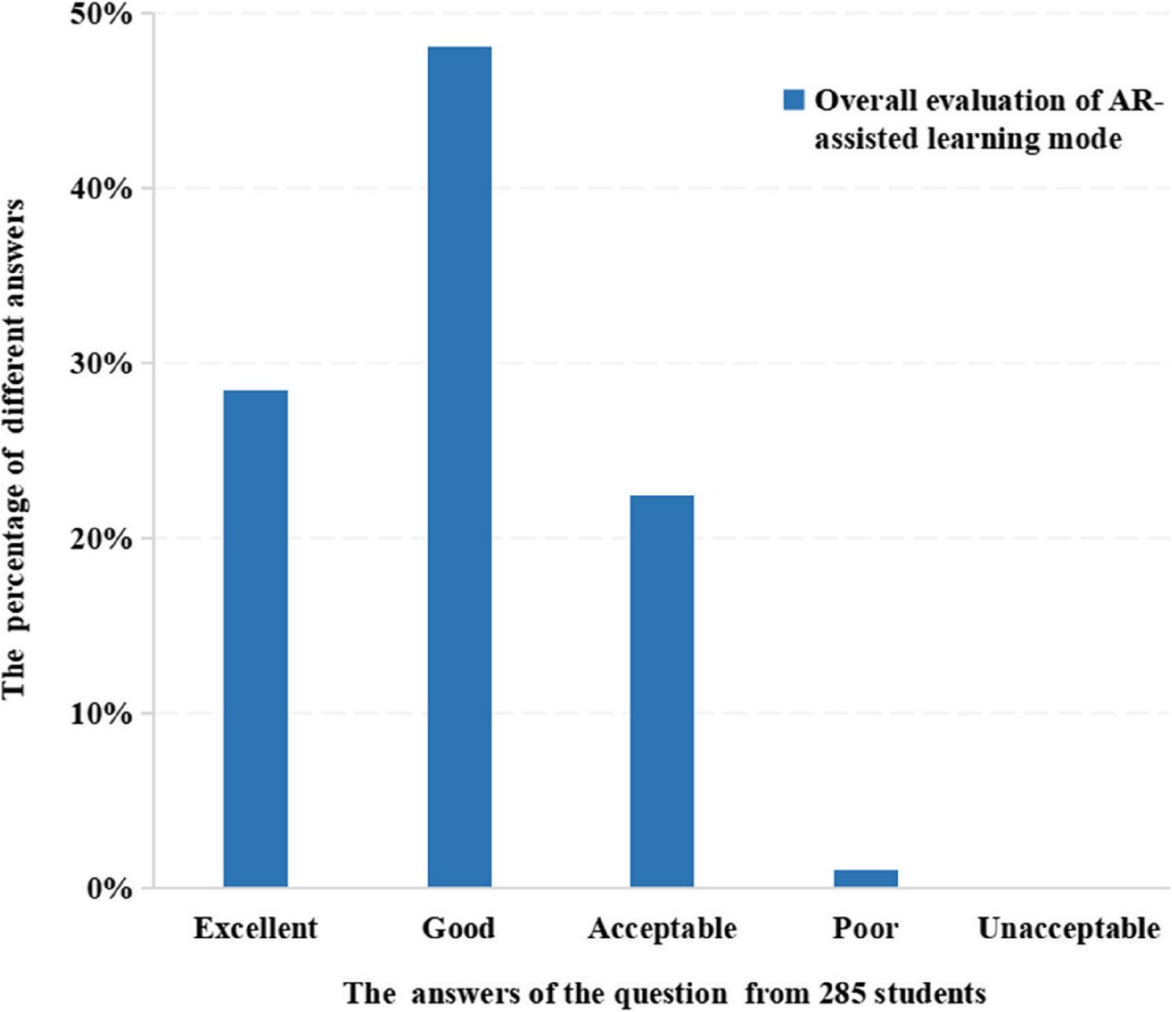
The distribution of different answers of the overall evaluation on AR-assisted learning model from 285 students including excellent to unacceptable

AR-assisted learning could be categorized into pre-class learning and post-class learning. Interestingly, compared with pre-class learning, post-class learning received greater acceptance among students (26.32% vs.73.68%). Overall, the majority of students gave a positive evaluation (overall evaluation: excellent: 28.42%, good: 48.07%, acceptable: 22.46%), while only a few students gave the negative feedback for AR-assisted learning mode (1.05%) (Fig. 2).

### Satisfaction assessment for AR-assisted learning mode

In order to compare students’ attitudes toward the two teaching modes, a hundred-mark system was used to score them. For the proportion of satisfaction (score ≥ 60 points), our results showed that there was no significant difference between the AR-assisted learning and traditional teaching mode neither for the female reproductive system anatomy Sect. (99.65% vs. 99.65%, χ^2^ = 0.00, *p* = 1.00), or the normal delivery Sect. (99.65% vs. 99.65%, χ^2^ = 0.00, *p* = 1.00).

Based on the feedback from the students (Table 2), we found that top advantages of AR-assisted learning mode was clearer, more intuitive and vivid (56.14%), which could strengthen the memory of important knowledge. Other perceived benefits included enhanced spatial understanding (9.47%), flexibility in time or place (2.81%), and increased learning interest (2.11%). However, the students also identified areas for improvement such as complex and time-consuming use process and non-exquisite 3D pictures (22.11%), inconvenience of needing books and electronic devices (5.61%), and lack of more additional functions such as voice or text explanation, videos and note (3.51%).

**Table 2:** The advantages and disadvantages of AR-assisted learning mode assessed by the students

Specific evaluation		*N* = 285	*N*%
Advantages	Clearer, more intuitive and vivid	160	56.14%
	Increased understanding of the spatial structure	27	9.47%
	Convenience and not limited by time or place	8	2.81%
	Increased learning interest	6	2.11%
	NA	84	29.47%
Disadvantages	Complex and time-consuming use process, and non-exquisite 3D pictures	63	22.11%
	Inconvenience of needing books and electronic devices	16	5.61%
	Lack of additional functions such as voice or text explanation, videos and note	10	3.51%
	High memory consumption for AR software downloads	3	1.05%
	NA	193	67.72%

*NA meant some advantages or disadvantages were not applicable since some students did not provide specific evaluation for AR-assisted learning mode

## Discussion

As an emerging technology, AR technology has been increasingly explored across disciplines and applied in diverse educational scenarios [17–22]. For instance, AR-based softwares such as HoloHuman [17], Microsoft HoloLens2 [23], and MagicMirror [20] have been successfully developed for anatomical teaching. The application of the AR ultrasonography has demonstrated efficacy in enhancing students’ clinical diagnostic skills [19]. Given the challenges students face in understanding the pelvic anatomy and delivery process through traditional teaching mode, AR technology holds significant promise for obstetrics and gynecology (OBGY) education. This study is the first to demonstrate that AR technology could effectively stimulate learning interest, improve learning engagement and enhance the teaching quality in OBGY education for medical undergraduates.

Our findings showed that most students spent no more than 20 min for each AR-assisted learning section, suggesting minimal additional time burden compared to traditional methods. Kugelmann et al. demonstrated that the AR Magic Mirror system was a very useful addition to the gross anatomy course among 880 students due to the interactive nature of the system [20]. Moro C et al. also found AR learning mode was non-inferior to tablet-based learning mode while fostering deeper learner immersion and engagement among 59 participants [21]. Additional studies highlighted AR’s unique advantages in clinical skill training such as visualization and reproducibility in digital rectal examination and central venous catheterization [24, 25] and greater knowledge acquisition [26, 27]. Our study further verified that AR-assisted learning mode could effectively stimulate learning interest and promotes active engagement in OBGY education. As motivation is a key driver of learning [28], AR’s capacity to enhance knowledge retention, train clinical thinking and reduce the learning difficulty is biologically plausible and supported by our results. In particular, the improvement of clinical thinking ability facilitated by AR technology has mainly been based on students’ subjective evaluations. We hypothesized that the primary mechanism may lie in AR’s ability to help students understand 3D spatial structures, especially the delivery process, which is crucial for the diagnosis of abnormal labor. Notably, students perceived AR as a tool that enabled flexible use of spare time for pre-class preview and post-class review, leveraging its accessibility compared to physical anatomical specimens. Interestingly, a striking majority (73.68%) preferred post-class AR learning over pre-class use, which may reflect that AR can enhance learning outcomes after students have acquired preliminary theoretical knowledge.

Regarding satisfaction, no significant difference was observed between AR and traditional teaching modes, underscoring that the quality of traditional teaching mode is reliable in our hospital. To our surprise, almost all students (98.95%) provided positive feedback on AR-assisted learning mode in our study, similar to the 95.6% satisfaction rate reported for AR Magic Mirror in gross anatomy teaching (95.6%) [19]. This highlights AR’s exceptional potential and value to complement traditional teaching in OBGY education.

Considering more irreplaceable advantages in AR technology, AR-assisted learning mode might be superior to the traditional teaching mode. However, there were still some dissatisfied aspects for current AR technology. For instance, the AR software exhibited cumbersome usability, limited functionality, and suboptimal 3D image quality. To address these, future developments could include interface simplification, voice-guided narration, screenshot capabilities, etc. In addition, integrating AR into post-class case-based exercises may further reinforce knowledge retention, aligning with students’ preference for post-class AR use (73.68%).

To our knowledge, this is the first study to investigate AR integration in OBGY education, providing preliminary evidence for the establishment and promotion of AR-assisted learning in the future. Nevertheless, it still has some limitations. Firstly, as this study aims to pilot the feasibility of AR technology in OBGY teaching through evaluating student perceptions, objective evaluations of clinical thinking improvement, learning efficiency and AR model validity were not included. Secondly, the present study did not compare groups. Therefore, the actual efficacy of AR-assisted learning cannot be adequately evaluated. However, due to the potential value of AR in anatomy learning, future research would employ randomized controlled trials and incorporate quantitative outcomes to rigorously assess the learning effects of using AR technology in OBGY teaching.

## Conclusion

Our study demonstrated that AR technology significantly enhanced students’ learning engagement and facilitated a comprehensive understanding of the spatial anatomical structure. This technology is worthy to be integrated into traditional OBGY teaching as an additional learning tool for medical undergraduates. With continuous advancements in AR technology, we believe that the standardized AR-assisted learning protocols for OBGY would be established eventually, and the AR-assisted learning mode would become an important resource in the modern medical education.

## Supplementary Information

Supplementary Material 1.

## Data Availability

The primary data are available from the corresponding author on reasonable request.
