# Supplementary material for: Application of augmented reality technology for obstetrics and gynecology teaching among undergraduates
Source: BMC Med Educ. 2025 Aug 8;25:1151. doi: 10.1186/s12909-025-07751-1 (PMC12333177; doi:10.1186/s12909-025-07751-1)
Supplement: Supplementary file 1 — Supplementary Material 1. [file 12909_2025_7751_MOESM1_ESM.docx]

**Supp_Table 1.** Questionnaire provided to the students, containing explicit statements and evaluation about AR-assisted learning mode (without answers).

| **Questions** |
| --- |
| How long did it take you to learn the course on anatomy of the female reproductive system by AR？（minute） |
| How long did it take you to learn the course on anatomy of delivery process by AR？（minute） |
| You prefer AR-assisted learning mode |
| AR helps stimulate learning interest |
| AR helps promote active learning |
| AR helps understand the three-dimensional spatial structure |
| AR helps improve memory of learning content |
| AR helps train clinical thinking |
| Do you think there is a difference in the learning effect between the AR-assisted pre-class and after-class learning? |
| Your overall evaluation of AR-assisted learning mode |
| Rating your satisfaction with the AR-assisted course on anatomy of the female reproductive system (points) |
| Rating your satisfaction with the AR-assisted course on anatomy of the female reproductive system (points) |
| Rating your satisfaction with the AR-assisted course on anatomy of delivery (points) |
| Rating your satisfaction with the AR-assisted course on delivery (points) |
| Write down the advantages and disadvantages of AR-assisted learning mode |
| Write down the suggestions for improvement of AR-assisted learning mode |
